# Differences in public and producer attitudes toward animal welfare in the red meat industries

**DOI:** 10.3389/fpsyg.2022.875221

**Published:** 2022-09-12

**Authors:** Grahame J. Coleman, Paul H. Hemsworth, Lauren M. Hemsworth, Carolina A. Munoz, Maxine Rice

**Affiliations:** Faculty of Veterinary and Agricultural Sciences, Animal Welfare Science Centre, The University of Melbourne, Parkville, VIC, Australia

**Keywords:** general public, livestock producer, farm animal welfare, attitudes, trust, knowledge

## Abstract

Societal concerns dictate the need for animal welfare standards and legislation. The public and livestock producers often differ on their views of livestock welfare, and failure to meet public expectations may threaten the “social license to operate” increasing the cost of production and hampering the success of the industry. This study examined public and producer attitudes toward common practices and animal welfare issues in the Australian red meat industry, knowledge of these practices, and public and producer trust in people working the red meat industry using an Australia-wide survey of both the general public (*n* = 501) and red meat producers (*n* = 200). Public participants were recruited using a random digit dialing telephone survey (Computer-Assisted Telephone Interviewing) while the red meat producers were randomly selected within a curated database of Australian red meat producers. After controlling for gender and age, there were marked differences (*p* < 0.01) between public and producer respondents in 20 of the 27 attitude, trust and knowledge variables studied. Producers reported more positive beliefs in the conditions provided for sheep and beef cattle during sea and land transport, the husbandry practices used in the red meat industry, and red meat attributes regarding human health, environmental impact, animal use and animal welfare. Both public and producers reported similar levels of trust in conventional and commercial media and had similar beliefs about animal rights, prevention of animal cruelty and balancing the welfare of people and animals. The results indicate a polarization between the public and livestock producers in their attitudes toward animal welfare, knowledge of husbandry practices and trust in livestock people.

## Introduction

Public concern about the welfare of animals used by humans is not a new phenomenon and has continued to increase in importance during the last century (Alonso et al., 2020). The public is often a key driver of farm animal welfare change since public views affect decision makers at the political, regulatory, retail and industry levels (Coleman, 2018). Societal concerns dictate the need for animal welfare standards and animal welfare legislation, maintaining public trust allows the industry to operate with more flexibility and self-regulation of these standards, this is termed “social license to operate” (Arnot, 2009). Failure to meet the expectations of the public can lead to “social control” with increased consumer demands for litigation, regulations, and bureaucratic compliance, all of which increase the costs and hamper the success of the livestock industry (Rollin, 2004; Arnot, 2009). While consumers, as well as stakeholders in the meat supply value chain affect the supply and market shares of animal food products, they also affect the animal welfare standards, some of which can often exceed legislative requirements (Vanhonacker and Verbeke, 2014).

According to the Theory of Planned Behavior, behavior is a function of three factors: (1) attitudes toward the behavior, (2) perceived social pressure otherwise known as subjective norms, and (3) perceived behavioral control (Ajzen, 1991). Beliefs are “subjective facts” which form a major component of the attitudes or learned dispositions, which in turn have a role in determining how people behave as consumers and as citizens (Coleman, 2010). The term “consumers” refers to those who acquire and consume animal products while the term “citizen” refers more broadly to those who may not consume animal products, but nevertheless engage in behaviors that impact animal industries. These behaviors include purchasing behavior as well as other behaviors that impact the animal industries, including acts of public support of or opposition to the industries. Public attitudes to farm animal welfare appear to be based on limited knowledge (Coleman et al., 2015), and there is no correlation between perceived knowledge and actual knowledge of husbandry practices, for example (Coleman, 2018). Malek et al. (2018) assessed actual knowledge in an Australian sample, but did not report the correlations between attitudes and knowledge. However, they did report that those with the most negative attitudes to farm animal welfare also had the lowest actual and perceived knowledge. The public’s beliefs are largely acquired from the mass media, often filtered by opinion leaders (Coleman, 2018; Coleman et al., 2018, Malek et al., 2018). Attitudes to farm animal welfare form only part of the multifaceted factors that motivate consumer behavior (see, for example, Curtis et al., 2011). These attitudes are usually studied with a view to understanding consumer behavior, the polarization in views between animal activists and those who farm animals, and to gauge public perceptions regarding the use of animals (Coleman et al., 2015; Coleman, 2018).

Generally, research does not link public attitudes to any behaviors other than purchasing and it is known that purchasing is largely driven by concerns about food quality, health and cost (Curtis et al., 2011). Several studies have investigated public and livestock producer attitudes toward the welfare of livestock in Australia (Coleman and Toukhsati, 2006; Coleman et al., 2016, 2018). Views of what is important for livestock welfare often differ widely between the public and producers (Buddle et al., 2021), with intensification of animal production, freedom to move, social interactions and similar issues being prominent themes in community concerns [e.g., Sørensen and Fraser (2010) and Coleman et al. (2016)], whereas meeting basic health requirements has been reported as the primary focus for producers (Te Velde et al., 2002; Bock and van Huik, 2007; Vanhonacker et al., 2008; Spooner et al., 2012). Despite the differences between the two groups, livestock producer attitudes to animal welfare have typically received less attention than those of the general public (Buddle et al., 2021) and largely has involved qualitative interviews with smaller producer samples (e.g., Te Velde et al., 2002; Buddle et al., 2021). It is desirable to augment the findings from this qualitative research using larger scale quantitative studies to provide more comprehensive results.

Understanding public and livestock producers’ attitudes toward the welfare of livestock is important. For example, understanding producers’ attitudes can be used by governments and industries in developing animal welfare policy, while understanding public attitudes can assist in developing strategies for managing public perception (concerns and attitudes) in the broader community (Coleman et al., 2016). Beliefs influence perception and have a role in determining how people behave both as consumers and as citizens. Differences in the views of producers compared to the general public can have an impact on the commercial viability and sustainability of the animal industries (Coleman, 2010). Therefore, if the objective is to reduce the disparity between public and producer perceptions, possible strategies to achieve convergence between public and producer attitudes to livestock welfare include: increasing transparency to the public in farming techniques and a clear articulation of the implications of a farming practice on animal welfare as well as animal health, food safety and environmental impact; developing a shared understanding of what current practices are and what science can reveal about welfare; and the fostering of a culture shift among the livestock industries to sensitize them to changing cultural values about animal quality of life (Coleman, 2010, 2018). However, these strategies require evidence-based evaluation. Interventions to achieve a convergence in attitudes between producers and the general public can be based on an understanding of attitudes to animal welfare issues in a specific livestock industry. Yzer (2013) has proposed that the relevant attitudes to target are the behavioral, normative and control beliefs about the behaviors that people engage in that are motivated by animal welfare concerns.

Animal welfare relevant behaviors of both the public and livestock producers can affect commercial viability and even the sustainability of animal industries. Livestock producers’ attitudes to animal welfare affect animal welfare practices on farm. These practices include such things as the use of pain relief for husbandry procedures and stock handling. In the case of the general public, purchasing behavior and public expressions of concern about farm animal welfare in the form of, for example, public protests, writing to newspapers or politicians, social media posts etc., are welfare-related behaviors (Coleman et al., 2015). As discussed earlier, the public is often a key driver of animal welfare change since public views affect decision makers at the political, regulatory, retail and industry levels. Societal concerns dictate the need for animal welfare standards and animal welfare legislation, however, public attitudes about animal-use and animal welfare are often based on limited knowledge of industry practices and the reasons for them, and the public’s beliefs are largely acquired from the mass media and opinion leaders (Coleman et al., 2015).

The present study is part of a long-term project examining communication strategies to achieve a degree of convergence in attitudes between the general public and red meat producers, a greater awareness of each group’s concerns and improved trust. The aims of the present study were to survey both the general public and red meat producers in order to 1. understand public and producer attitudes toward, and knowledge of, common practices and animal welfare issues in the red meat industry, and 2. to understand public and producer trust in livestock people in the red meat industry.

## Materials and methods

### Questionnaire development

The questionnaire used for the surveys was based on those developed by the Animal Welfare Science Center, University of Melbourne to study public attitudes and knowledge of animal use and welfare in a range of livestock industries including the pork, egg and red meat industries (Coleman and Toukhsati, 2006; Coleman et al., 2016, 2018). The questionnaire was modified to meet the specific objectives of the present study by targeting issues in the red meat industries, was reviewed by members of the project’s advisory committee, comprising key stakeholders from the sheep industry, beef cattle industry, Meat Livestock Australia, RSPCA Australia and Animal Welfare Science Center and was piloted on a range of researchers, red meat industry personnel and members of the general public. The final questionnaire comprised five sections (see Table 1). Question formats included dichotomous (e.g., yes or no), multiple choice, and Likert scale which were used to measure the participant’s agreement with statements about their opinions, behaviors and knowledge with regard to farm animals and the livestock industries (e.g., “It is appropriate to use sheep and beef cattle to produce food for humans”). Some questions were asked separately in relation to the two species, however in the interest of keeping the questionnaire to an acceptable duration this could not be done for all questions. Previous research suggests that public attitudes toward beef and sheep welfare are similar (Coleman et al., 2019), thus the decision was made to combine the two for the majority of the questions.

**TABLE 1:** Questionnaire structure used for both the public and the producer questionnaires.

Section	Number of questions	Type of information gathered
Demographics	11	Age, gender, education, location, dietary preferences and farm demographics (in producer questionnaire)
Animal welfare	8	General attitudes toward animal welfare, trust of people involved in farm animal production, normative and control beliefs in relation to animal welfare
Knowledge of farm animals and farm animal welfare	14	Perceived and actual knowledge of beef cattle and sheep production practices (e.g., ear tagging, mulesing, vaccination, crutching etc.)
Attitudes toward red meat farming	6	Approval of red meat farming practices, importance of social contact, nutrition, shelter, medications etc., concern about land and sea transport conditions.
Behavior in relation to farm animal welfare	8	Membership to Animal rights groups, community behaviors (e.g., volunteering, writing letters to politicians), sources of information about animal welfare, conversations about animal welfare

### Data collection method

I-View, a specialized market and social research data collection agency, was contracted to deliver the questionnaire to a convenience sample of the general public using random telephone recruitment (Computer Assisted Telephone Interview, CATI) of 501 participants. CATI involved dialing random fixed-line and mobile telephone numbers and inviting potential participants to complete the questionnaire by telephone. In each call, the consultant initially requested the youngest male over the age of 18 years in the household in order to counteract the expected bias for older female participants. Initially, only landline numbers were used, however despite asking for the youngest male in the household, there was still a considerable bias toward older females. To counteract this bias, iView began including mobile numbers and targeted areas with more youthful demographics. The questionnaire was delivered over 26 days in March-April 2018. A specialist agriculture market research company, Kg2, was contracted to deliver the questionnaires to red meat (beef cattle and sheep) producers. The questionnaires were delivered using the CATI method to a convenience sample of producers randomly selected within a curated database of approximately 200,000 contacts covering 80 farm types. The questionnaire was delivered to 200 producers over 30 days in September–October.

### Statistical analyses

Statistical analyses were performed using the statistical package SPSS 25.0 (SPSS Inc., Chicago, IL, United States). The attitude and trust questionnaire data comprised a series of Likert scale questions asking about participants’ agreement with statements reflecting their general attitudes toward animal welfare, attitudes toward using animals and attitudes toward the Australian red meat livestock industry (e.g., “It is appropriate to use sheep and beef cattle to produce food for humans” and “I trust farmers to properly care for their sheep and beef cattle”). These data, for the combined public and producer respondents, were analyzed using Principal Components Analysis (PCA) followed by either a Varimax or an Oblimin rotation to identify commonalities amongst the questionnaire items. The Varimax or Oblimin rotations were performed on component solutions of more than one component to provide the best simple structure (Tabachnick and Fidell, 2012). The suitability of the data for the analysis was assessed using criteria outlined by Pallant (2013); the correlation coefficients were all above the required 0.3, the Kaiser-Meyer-Olkin (KMO) values exceeded the recommended value of 0.6, and Bartlett’s Test of Sphericity reached statistical significance. Before conducting the PCAs, items were recoded where appropriate so that high scores reflected positive attitudes, high trust, etc. Items that were established as belonging to a common underlying component were then averaged to produce a composite score for that component. The data from the general public using this questionnaire have previously been published by Rice et al. (2020) and Hemsworth et al. (2021). Initially, PCA analyses were carried out on each sample separately to determine if the solutions were similar. Where differences were observed, items were removed until the structures were similar. Following this, the PCAs were carried out on the combined samples (see Table 2). This procedure resulted in some subtle differences in the components from those reported in Rice et al. (2020) and Hemsworth et al. (2021) and, therefore, the resulting composite scores.

**TABLE 2:** Components from the questionnaire grouped into composite scores, with a high score (on a 5-point scale) indicative of a positive attitude to or strong agreement with the statements (questionnaire items).

Topic	Assigned attitude component label	Cronbach’s Alpha	Questionnaire item	Loadings
Meaning of animal welfare	Animal welfare -humane	0.76	Preventing animal cruelty	0.98
			Humane treatment of animals	0.77
	Animal welfare – handling	0.77	Farmers and farm animal handlers using best practice	−0.93
			Farmers and farm animal handlers caring for their animals	−0.84
	Animal welfare -people vs animals	0.49	Balancing the needs of animals and people	0.92
			Caring for our pets	0.66
Acceptability of animal uses	Red meat attributes	0.84	I believe beef and lamb are healthy foods	0.83
			It is appropriate to use sheep and beef cattle to produce food for humans	0.81
			Sheep and beef cattle farming is environmentally sustainable	0.82
			Sheep and beef cattle are raised in a humane and animal friendly manner	0.79
	Red meat animal rights	0.59	Sheep and beef cattle have the same right to life as domestic animals	0.84
			Sheep and beef cattle have the same feelings as domestic animals	0.83
Behavioral beliefs	Public engagement beliefs	0.83	It is important for me to encourage family and friends to be actively involved in the promotion of animal welfare	0.88
			I should encourage my friends to support animal welfare causes	0.84
			I think it is important to lobby governments to improve the welfare of farm animals	0.72
			It is important for me to be actively involved in the promotion of farm animal welfare	0.76
Normative beliefs	Negative normative beliefs	0.70	Lobbying the government to improve the welfare of farm animals is not something my partner/family would expect me to do	−0.78
			My partner/family would not expect me to encourage my family and friends to be actively involved in the promotion of animal welfare	−0.86
			The welfare of farm animals is not something that my partner/family would expect me to consider when making meat shopping choices	−0.70
	Positive normative beliefs	0.73	My partner/family would expect me to buy lamb and beef that is produced with good animal welfare practices	0.85
			My partner/family would expect me to encourage my friends to support animal welfare causes	0.74
			My partner/family would expect me to be actively involved in the promotion of farm animal welfare	0.74
Control beliefs	Difficult to act	0.39	I find it takes too much effort to buy beef and lamb that is produced with good animal welfare practices.	0.82
			I would find it too difficult to lobby the government to improve the welfare of farm animals	0.73
	Easy to act	0.72	I can easily encourage my friends to support animal welfare causes	0.88
			I can easily be involved actively in the promotion of farm animal welfare	0.85
Trust of livestock industry people	Trust livestock people	0.92	I trust farm animal handlers to properly care for their sheep and beef cattle	0.92
			I trust those responsible for transporting sheep and beef cattle by land to properly care for them	0.90
			I trust abattoir workers who work with sheep and beef cattle to properly care for them and use humane slaughter methods	0.89
			I trust farmers to properly care for their sheep and beef cattle	0.88
Attitudes toward red meat farming practices	Approval of husbandry practices	0.89	Castration	0.81
			Crutching	0.76
			Tail docking	0.75
			Dehorning	0.74
			Ear tagging	0.74
			Feedlotting	0.68
			Curfew	0.65
			Hot iron branding	0.61
			Pre-slaughter stunning	0.62
			Spaying	0.53
			Mulesing	0.52
Importance of farming attributes	General welfare	0.80	Regular exercise	0.73
			Freedom to roam outdoors	0.81
			Social contact with animals of the same species	0.73
			Good nutrition	0.65
			Fresh air	0.71
			Access to water	0.55
			Contact with their young	0.57
			Shelter	0.53
			Protection from predators	0.46
			Pain relief during painful husbandry procedures	0.34
	Medication	0.73	Medications (i.e., antibiotics) for health	0.84
			Vaccinations for health	0.87
Comfort of beef cattle	Land beef transport conditions	0.94	Space per animal	0.90
			Journey length	0.90
			Road/truck conditions (e.g., sound, vibration, braking levels	0.90
			Provision of food and water	0.79
			Ventilation	0.86
			Loading of animals onto vehicles (e.g., use of handling aids, human handling)	0.79
	Sea beef cattle transport conditions	0.96	Space per animal	0.94
			Ventilation	0.94
			Boat conditions (e.g., sounds, vibration, unsteady ground)	0.92
			Provision of food and water	0.91
			Journey length	0.90
			Loading of animals onto boats (e.g., use of handling aids, human handling)	0.88
Comfort of sheep	Land sheep transport conditions	0.96	Space per animal	0.94
			Journey length	0.94
			Road/truck conditions (e.g., sound, vibration, braking levels	0.94
			Provision of food and water	0.83
			Loading of animals onto vehicles (e.g., use of handling aids, human handling)	0.91
			Ventilation	0.91
	Sea sheep transport conditions	0.97	Space per animal	0.95
			Ventilation	0.95
			Boat conditions (e.g., sounds, vibration, unsteady ground)	0.95
			Journey length	0.91
			Provision of food and water	0.92
			Loading of animals onto boats (e.g., use of handling aids, human handling)	0.89
Accessing information	Conventional media	0.74	Print media (e.g., magazines, newspapers, scientific papers)	0.77
			Radio	0.76
			Television (e.g., TV news, documentaries)	0.66
			Industry bodies	0.64
			Government advertisements/promotions	0.55
	Social and internet media	0.69	Social network sites, related social media (e.g., Facebook, YouTube, Twitter, blogs)	0.83
			Internet	0.85
			Friends, relatives or colleagues	0.50
			Animal welfare organizations e.g., RSPCA	0.55
	Commercial media	0.63	Supermarkets (e.g., Coles, Woolworths, IGA)	0.82
			Celebrity chef/cook	0.69
			Labels (product labels)	0.63
Trust of information sources	Trust social and internet media	0.80	Television (e.g., TV news, documentaries)	0.76
			Animal welfare organizations e.g., RSPCA	0.76
			Internet	0.74
			Radio	0.71
			Social network sites, related social media (e.g., Facebook, YouTube, Twitter, blogs)	0.67
			Print media (e.g., magazines, newspapers, scientific papers)	0.64
	Trust conventional and commercial media	0.77	Industry bodies	0.83
			Supermarkets (e.g., Coles, Woolworths, IGA)	0.80
			Labels (product labels)	0.69
			Government advertisements/promotions	0.59
			Celebrity chef/cook	0.46

Cronbach’s Alpha was calculated using the full sample.

Scale reliabilities were measured using Cronbach’s α coefficients with an α = 0.70 as the criterion for acceptable reliability (DeVellis, 2003). Items were included in a scale if their loading on the relevant component exceeded 0.33 (Tabachnick and Fidell, 2012) and if, on the basis of face validity, they could be summarized by just one construct. A summary of the details of the component structures and Cronbach’s α coefficients are reported in Table 2. As can be seen in this table, most Cronbach’s α coefficients exceeded 0.7 with the exception of five components labeled Animal welfare people/animals; Red meat animal rights; Easy to act; Commercial media; and Social and internet media. In three of the five components, only two items comprised the composite score and Cronbach’s α coefficients are generally low where there are few items in the scale (Nunnally et al., 1967).

There were two sets of questions relating to knowledge of the red meat industry. Perceived knowledge was measured by asking respondents “How much do you feel you know about beef cattle production?” and “How much do you feel you know about sheep production?” Actual knowledge was assessed through a series of 13 multiple choice questions in relation to some common farming practices, with three questions specific to sheep husbandry (e.g., mulesing, crutching, tail docking) and three questions specific to beef cattle husbandry (e.g., dehorning, branding, spaying), and the remaining questions related to practices that are common to both sectors (e.g., pre-slaughter stunning, ear tagging, curfew, feedlotting, castration, use of growth hormones, use of antibiotics). Respondents were then given a score (knowledge score) based on the proportion of correctly answered questions. Respondents were also asked about their level of concern for both the welfare of sheep and of beef cattle, and these questions were answered using a 5-point Likert scale from “extremely concerned” to “not concerned.”

Community behaviors, which were defined as behaviors specifically performed to express dissatisfaction with any aspect of sheep or cattle farming and varying in degrees of anonymity, effort and commitment, were assessed on a dichotomous scale as to whether the participants had or had not previously performed the behavior. There were 10 behaviors in total, ranging from discussions with friends, colleagues and family through to attending a public rally or demonstration. The sum of the community behaviors (possible range 0–10) was used for analysis.

Analysis of covariance was used to examine the effects of type of respondent (public and producer) and gender, with age as a covariate, on the composite variables. In this analysis of each composite variable, if the dependent variable failed the test of homogeneity of variance (based on Levene’s test), a more stringent test of the main effects was adopted, that is, *p* < 0.01 instead of *p* < 0.05 (Tabachnick and Fidell, 2012).

## Results

The average duration of the CATI survey for the public was 36 min and for the producers 31 min, and response rates were 15 and 18%, respectively. A summary of the age/gender demographics from the survey is presented in Table 3. In relation to the 200 producers surveyed, there were 52 sheep producers, 81 beef producers, 65 sheep and beef producers and 2 other producers (one dairy producer selling calves for meat, and one sheep/goat producer).

**TABLE 3:** Age/gender demographics of the public and red meat producer respondents (Census data in italics where available).

	Public			Producer		
Age	% Male	% Female	Total	% Male	% Female	Total
18–24	40	60	48	50	50	4
25–34	47 *(49)*	53 *(51)*	75	86	14	7
35–44	54 *(49)*	46 *(51)*	84	86	14	14
45–54	39 *(49)*	61 *(51)*	96	73	28	40
55–64	53 *(49)*	47 *(51)*	94	74	26	65
65+	43 *(46)*	56 *(54)*	104	76	24	70
Overall	46 *(49)*	54 *(51)*	501	75	25	200

### Comparison of public and producer attitudes, trust, and knowledge

As shown in Table 4, there were marked differences (at *p* < 0.01) between public and producer respondents in 20 of the 29 attitude, knowledge and trust variables studied. Based on the adjusted mean, producer attitudes in general were more positive than those of the public with the public attitudes being for the most part negative toward transport of sheep and beef cattle. In particular, producers reported more positive beliefs in the conditions provided for sheep and beef cattle during sea and land transport; the husbandry practices used in the red meat industry; and red meat attributes regarding human health; environmental impact; animal use and animal welfare, and also in their belief that animal welfare involves livestock people caring for their animals and using best practice. In addition, producers also reported less concern about beef cattle and sheep welfare than the public.

**TABLE 4:** The effects of type of respondent (public and producer) and gender, with age as a covariate, on the composite variables.

	Adjusted mean (std error)						*P*-value			Covariate
	Public			Producer			Type	Gender	Interaction	*P*-value
Composite variable	Overall	Male	Female	Overall	Male	Female				
Sea beef transport conditions	2.12 (0.05)	2.28 (0.07)	1.96 (0.06)	3.65 (0.09)	3.8 (0.09)	3.5 (0.15)	<0.01*^c^*	<0.01*^a^*	0.94	0.42
Land sheep transport conditions	2.37 (0.05)	2.53 (0.07)	2.21 (0.06)	3.91 (0.09)	3.98 (0.09)	3.85 (0.15)	<0.01*^c^*	0.02	0.31	0.23
Sea sheep transport conditions	2.03 (0.05)	2.17 (0.07)	1.89 (0.07)	3.48 (0.09)	3.66 (0.09)	3.29 (0.15)	<0.01*^c^*	<0.01*^a^*	0.62	0.57
Beef cattle welfare concern	2.54 (0.06)	2.76 (0.08)	2.32 (0.08)	4.22 (0.1)	4.41 (0.1)	4.02 (0.18)	<0.01*^c^*	<0.01*^a^*	0.86	0.06
Land beef transport conditions	2.53 (0.04)	2.68 (0.07)	2.37 (0.06)	3.87 (0.08)	3.93 (0.08)	3.81 (0.14)	<0.01*^c^*	0.02	0.29	<0.01*^a^*
Sheep welfare concern	2.56 (0.05)	2.74 (0.08)	2.37 (0.07)	4.17 (0.1)	4.31 (0.1)	4.02 (0.17)	<0.01*^c^*	<0.01*^a^*	0.71	<0.01*^a^*
Approval of husbandry practices	3.06 (0.04)	3.23 (0.05)	2.9 (0.05)	4.05 (0.07)	4.07 (0.07)	4.03 (0.12)	<0.01*^c^*	0.02	0.06	0.03
Knowledge Score	72.77 (0.72)	72.82 (1.05)	72.71 (0.97)	92.02 (1.32)	92.45 (1.32)	91.59 (2.25)	<0.01*^c^*	0.74	0.80	<0.01*^a^*
Red meat attributes	3.69 (0.04)	3.78 (0.06)	3.59 (0.05)	4.63 (0.07)	4.6 (0.07)	4.66 (0.12)	<0.01*^c^*	0.43	0.10	<0.01*^a^*
Trust	3.39 (0.05)	3.53 (0.07)	3.24 (0.06)	4.4 (0.09)	4.32 (0.09)	4.48 (0.15)	<0.01*^b^*	0.49	0.02	0.12
Perceived Knowledge beef	3.21 (0.05)	3.34 (0.07)	3.09 (0.07)	4.19 (0.09)	4.23 (0.09)	4.14 (0.15)	<0.01*^b^*	0.10	0.44	0.04
Trust social and internet media	2.96 (0.03)	2.89 (0.05)	3.04 (0.04)	2.48 (0.06)	2.45 (0.06)	2.51 (0.1)	<0.01*^b^*	0.12	0.45	<0.01*^a^*
Perceived Knowledge sheep	3.1 (0.05)	3.2 (0.08)	3 (0.07)	3.82 (0.1)	3.94 (0.1)	3.71 (0.17)	<0.01*^a^*	0.05	0.88	<0.01*^a^*
Conventional media	2.63 (0.04)	2.6 (0.06)	2.66 (0.06)	3.2 (0.08)	3.25 (0.08)	3.15 (0.13)	<0.01*^a^*	0.74	0.33	<0.01*^a^*
Behavior	2.34 (0.08)	1.88 (0.12)	2.79 (0.11)	1.47 (0.15)	1.34 (0.15)	1.6 (0.25)	<0.01*^a^*	<0.01*^a^*	0.05	<0.01*^a^*
Commercial media	1.99 (0.03)	1.87 (0.05)	2.12 (0.04)	2.28 (0.06)	2.35 (0.06)	2.22 (0.1)	<0.01*^a^*	0.09	<0.01*^a^*	<0.01*^a^*
Animal welfare handling	4.28 (0.04)	4.21 (0.06)	4.35 (0.05)	4.63 (0.07)	4.59 (0.07)	4.66 (0.12)	<0.01*^a^*	0.20	0.68	0.28
Difficult to act	2.83 (0.05)	2.89 (0.07)	2.78 (0.06)	2.46 (0.09)	2.6 (0.09)	2.32 (0.15)	<0.01*^a^*	0.04	0.41	0.12
Medication	4.55 (0.03)	4.46 (0.04)	4.63 (0.04)	4.75 (0.06)	4.67 (0.06)	4.83 (0.1)	<0.01*^a^*	0.01*^a^*	0.98	0.57
General welfare	4.77 (0.02)	4.68 (0.02)	4.85 (0.02)	4.67 (0.03)	4.56 (0.03)	4.78 (0.05)	<0.01*^a^*	<0.01*^a^*	0.48	0.20
Easy to act	3.09 (0.05)	2.97 (0.08)	3.2 (0.07)	3.33 (0.1)	3.29 (0.1)	3.36 (0.16)	0.03	0.17	0.45	<0.01*^a^*
Positive normative beliefs	3.3 (0.05)	3.15 (0.07)	3.44 (0.07)	3.5 (0.09)	3.54 (0.09)	3.47 (0.15)	0.04	0.28	0.07	0.08
Trust conventional media	2.57 (0.04)	2.44 (0.05)	2.71 (0.05)	2.71 (0.07)	2.72 (0.07)	2.69 (0.11)	0.07	0.10	0.04	<0.01*^a^*
Public engagement beliefs	3.5 (0.05)	3.22 (0.07)	3.77 (0.07)	3.31 (0.09)	3.36 (0.09)	3.27 (0.16)	0.08	0.03	<0.01*^a^*	0.51
Social and internet media	2.65 (0.04)	2.46 (0.06)	2.85 (0.05)	2.53 (0.07)	2.41 (0.07)	2.65 (0.12)	0.14	<0.01*^a^*	0.35	<0.01*^c^*
Animal welfare humane	4.52 (0.04)	4.42 (0.05)	4.62 (0.05)	4.58 (0.07)	4.63 (0.07)	4.54 (0.11)	0.40	0.44	0.04	0.71
Animal welfare people animals	4.06 (0.04)	3.87 (0.06)	4.25 (0.06)	4.12 (0.08)	4.08 (0.08)	4.15 (0.13)	0.52	0.01*^a^*	0.07	0.15
Red meat animal rights	3.97 (0.05)	3.72 (0.07)	4.21 (0.06)	3.91 (0.08)	3.81 (0.08)	4.01 (0.14)	0.53	<0.01*^a^*	0.12	0.40
Negative normative beliefs	2.9 (0.05)	3.04 (0.07)	2.77 (0.07)	2.84 (0.09)	2.75 (0.09)	2.93 (0.16)	0.56	0.65	0.03	0.77

*^a^* Partial ETA squared <0.06; *^b^* Partial ETA squared 0.06–0.14; *^c^* Partial ETA squared >0.14. A high score for a composite variable is indicative of a positive attitude to or strong agreement with the questionnaire items in the composite variable (questionnaire items described in Table 2).

Producers reported that it was easier to lobby and promote animal welfare than the public (X̄Adj_*Public*_ 3.09, X̄Adj_*Producer*_ 3.33; *p* = 0.03). Furthermore, the public reported that it was more difficult to purchase red meat produced under good welfare standards and to lobby governments to promote farm animal welfare than producers (X̄Adj_*Public*_ 2.83, X̄Adj_*Producer*_ 2.46; *p* < 0.01). However, producers reported stronger beliefs that that people that matter to them expected them to both purchase lamb and beef produced with good welfare, support animal welfare causes and lobby governments to improve animal welfare than the public (X̄Adj_*Public*_ 3.3, X̄Adj_*Producer*_ 3.5; *p* = 0.04).

In comparison to the public, producers reported higher levels of trust in livestock people (farmers and handlers) caring for their animals (X̄Adj_*Public*_ 3.39, X̄Adj_*Producer*_ 4.40; *p* < 0.01), but reported lower levels of trust in social and internet media (X̄Adj_*Public*_ 2.96, X̄Adj_*Producer*_ 2.48; *p* < 0.01). Despite the reduced trust in social and internet media, producers reported similar use of social and internet media to the general public (X̄Adj_*Public*_ 2.65, X̄Adj_*Producer*_ 2.53; *p* = 0.14). Producers also reported more use of conventional media (X̄Adj_*Public*_ 2.63, X̄Adj_*Producer*_ 3.2; *p* < 0.01), and less use of commercial media (X̄Adj_*Public*_ 1.99, X̄Adj_*Producer*_ 2.28; *p* < 0.01). Producers also placed more importance on the use of medication (e.g., antibiotics) for health (X̄Adj_*Public*_ 4.55, X̄Adj_*Producer*_ 4.75; *p* < 0.01) and had greater perceived and actual knowledge (score) of sheep and cattle production (*p* < 0.01, see Table 4).

The public and producers reported similar levels of trust in conventional and commercial media (X̄Adj_*Public*_ 2.57, X̄Adj_*Producer*_ 2.71; *p* < 0.07). Furthermore, the public and producers had similar beliefs about the humane treatment of animals and preventing animal cruelty (X̄Adj_*Public*_ 4.52, X̄Adj_*Producer*_ 4.58; *p* = 0.40) and balancing the welfare of people and animals (X̄Adj_*Public*_ 4.06, X̄Adj_*Producer*_ 4.12; *p* = 0.52), and that sheep and beef cattle have similar rights and feelings as domestic animals (X̄Adj_*Public*_ 3.97, X̄Adj_*Producer*_ 3.91; *p* = 0.53).

There were some significant gender effects on the attitude, knowledge and trust variables as well as some significant interactions. Some of the notable gender effects were that males reported more positive beliefs in the conditions provided for sheep during sea transport (X̄Adj_*Male*_ 2.75, X̄Adj_*Female*_ 2.11; *p* < 0.01) and land transport (X̄Adj_*Male*_ 3.10, X̄Adj_*Female*_ 2.46; *p* < 0.01), and also had more positive beliefs in the conditions provided for beef cattle during sea transport (X̄Adj_*Male*_ 2.87, X̄Adj_*Female*_ 2.20; *p* < 0.01) and land transport (X̄Adj_*Male*_ 3.18, X̄Adj_*Female*_ 2.58; *p* < 0.01). In addition males showed more approval of the husbandry practices used in the red meat industry (X̄Adj_*Male*_ 3.56, X̄Adj_*Female*_ 3.07; *p* = 0.02), while females placed more importance on general welfare attributes (X̄Adj_*Male*_ 4.63, X̄Adj_*Female*_ 4.83; *p* < 0.01). Female respondents also reported more concern than males about beef cattle welfare (X̄Adj_*Male*_ 3.40, X̄Adj_*Female*_ 2.60; *p* < 0.01) and sheep welfare (X̄Adj_*Male*_ 3.35, X̄Adj_*Female*_ 2.65; *p* < 0.01).

## Discussion

In this study of Australian public and red meat producer attitudes there were some marked differences in the attitudes of the two groups toward aspects of red meat farming. Of all the attitudes studied here, the most marked differences between the public and producer attitudes were the attitudes toward the conditions under which sheep and beef cattle were transported, particularly during sea transport. Producers generally reported more positive beliefs about the conditions provided for sheep and beef cattle during sea and land transport while the attitudes of the public toward transport (both sea and land) of sheep and beef cattle were in general clearly negative. Similar findings were reported by Fleming et al. (2020) who found members of the general public had high levels of concern for the welfare of livestock in the live export industry, compared with low to no concern reported by respondents who worked in the live export industry. This generally negative attitude of the Australian public to livestock transport has been reported previously (Buddle et al., 2018; Sinclair et al., 2018; Fleming et al., 2020) and is not surprising since there have been several recent public media campaigns in Australia calling for bans to live export of Australian farm animals (Petrie, 2016; Buddle and Bray, 2019). Indeed, a very recent study of the Australian public found that a wide media coverage of live export of sheep by sea resulted in increased community discussion and social media activity as well as increased perceived importance of conditions aboard boats used for live sheep transport (Rice et al., 2020). Concern about the transport of livestock has been previously reported to rank highly amongst the public in Australia and Europe (Vanhonacker et al., 2010, 2012; Coleman et al., 2015).

Studies in Belgium and the Netherlands indicate that while livestock producers generally report a positive view of the welfare of livestock, the public and consumers have a more negative view of livestock welfare (Te Velde et al., 2002; Vanhonacker et al., 2008). In the present study, red meat producers reported generally positive beliefs about the husbandry practices used in the red meat industry while the public expressed neutral views. While the public in general neither agreed nor disagreed with the use of the main red meat husbandry practices, the attitude of producers was positive. Similarly, producers generally had positive attitudes to the notions that animal welfare involves both livestock people caring for their animals and using best practice, whereas the public in general neither agreed nor disagreed with these two notions. As has been reported previously, livestock producers had both greater perceived and actual knowledge (score) of livestock production practices than the public (Te Velde et al., 2002; Coleman et al., 2015; Lemos et al., 2018). These more positive producer attitudes toward transport of sheep and beef cattle, the husbandry practices commonly used in the red meat industry and the welfare implications of both livestock people caring for their animals and using best practice may therefore reflect their better knowledge of scientific and industry advice on managing and caring for their livestock, together with their better knowledge of the current Australian welfare standards and guidelines underpinning these practices, including sea and land transport of sheep and beef cattle (ANON, 2012, 2016a,2016b,^2020^) than the public. This is not to imply that factual knowledge is the only basis for welfare attitudes in general, nor that improving actual knowledge in the general public will, on its own, lead to more positive attitudes. However, because producers have a clear rationale for their beliefs that their practices have good welfare outcomes, they may be more generally positive in their attitudes. Furthermore, Buddle et al. (2021) reported that producers believe that their abilities to manage factors that impact animal welfare came with experience. While producers in general have first-hand knowledge of the management practices and commitment required in safeguarding animal welfare, there may also be elements of self-interest and defensiveness influencing some of these producer responses (Te Velde et al., 2002). The theory of cognitive dissonance suggests that people will rationalize a decision around opposing beliefs to reduce psychological stress and align their beliefs with their actions (Harmon-Jones and Mills, 2019). For a similar range of reasons as these, it is not surprising that producers reported more positive beliefs about red meat attributes regarding human health, environmental impact, animal use and animal welfare than the public. Producers also had higher levels of trust in livestock people (farmers and handlers) caring for their animals than the public. Coleman et al. (2015) found that while the Australian public in general reported some level of trust that livestock workers properly care for their animals, they expressed a low level of trust in sea and land transport workers to properly care for their animals. A similar result was found by Buddle et al. (2018) with members of the general public reporting lower levels of trust in livestock transporters when compared to their level of trust in producers. The positive view of producers that farmers care for their animals may reflect their knowledge of the management practices of other farmers, as well as recommendations arising from industry meetings and extension activities on the topic of safeguarding livestock welfare. In addition, these more positive attitudes of producers toward farm animal welfare could be a result of differences in the interpretation of animal welfare between producers and non-producers (Te Velde et al., 2002; Vanhonacker et al., 2008). An explanation for this difference could lie in the greater emphasis that producers reportedly place on biological functioning (Te Velde et al., 2002; Vanhonacker et al., 2008; Cantrell et al., 2013), while the public place more emphasis on affective states of the farm animals and naturalness in the consideration of animal welfare (Te Velde et al., 2002; Lassen et al., 2006; Vanhonacker et al., 2008; Prickett et al., 2010). Consequently because of the more positive attitudes of producers to transport and husbandry practices used in the red meat industry and the welfare implications of both livestock people caring for their animals and using best practice management, it is not surprising that, in the present study, producers generally reported less concern about beef cattle and sheep welfare than the public.

In comparison to the public, producers had higher levels of trust in conventional and commercial media, and not surprisingly made more use of conventional media. In contrast, producers had lower levels of trust in social and internet media than the public and consequently made less use of social and internet media. Producers also made less use of commercial media than the public. Less trust in and less use of social and internet media by producers may reflect their concerns that they may be confronted with criticism, and possibly of violent and insulting nature, using social media to discuss farming topics (Dürnberger, 2019). Increasing interest in animal welfare in Australia has reportedly led to farmers becoming the target of activism in the media, online, and through direct action (Mummery and Rodan, 2017; Moraro, 2019). These findings regarding trust and use of media sources are consistent with those found by Buddle et al. (2021) who reported that Australian red meat producers are concerned about the role of social media and the internet in spreading false information about livestock production in Australia.

There were differences between the public and producers in several control and normative beliefs. Producers reported that it was easier to support or promote positive animal welfare than did members of the general public, which may be a consequence of the greater knowledge and approval of the husbandry practices in the industry in producer respondents. Buddle et al. (2021) found that producers considered maintaining animal welfare to be important for productivity, so it may also be that they find it easier to promote positive animal welfare because they consider the safeguarding of animal welfare as part of their daily role. The greater belief by producers that it was easier to support or promote animal welfare, while perhaps not all that surprising, may also explain why producers were less concerned about animal welfare in the red meat industry. Interestingly though when considering gender, both female producers and members of the public reported a greater ability to lobby and promote animal welfare compared to male respondents. This finding indicates that females in general may feel more comfortable expressing their views on animal welfare. Although there is evidence that attitudes to livestock welfare is only one of the predictors of purchasing behavior with price, healthiness, and local production being more important for consumers (Coleman and Toukhsati, 2006; Coleman et al., 2015), public respondents reported that it is easier to purchase lamb and beef produced with good welfare than did producers. Public respondents also reported that it is easier to lobby governments to improve animal welfare than did producers, however, producers reported that people that matter to them expect them to do so more than did public respondents. These more positive control beliefs by the general public may reflect their greater commitment to express their concern about the welfare of beef cattle and sheep. Surprisingly, producers reported that people that matter to them expected them to both purchase lamb and beef produced with good welfare, support animal welfare causes and lobby governments to improve animal welfare than the public. It may be that because partners and family of producers are very closely associated with the red meat industry that they are more active in expressing the need to support or promote positive animal welfare than partners and family of public respondents.

The public and producers had similar attitudes to protecting the rights of animals, preventing animal cruelty and balancing the welfare of people and animals. This finding suggesting producers and the public share similar views regarding the importance of safeguarding sheep and beef cattle welfare is consistent with that reported by Vanhonacker et al. (2008), who found both groups had similar views on obtaining an acceptable level of farm animal welfare. Furthermore, the two groups had similar attitudes to the rights and feeling of sheep and beef cattle and that of domestic animals. However, although these groups share similar attitudes toward animal welfare, how they actually translate this into practice is likely to be the key. For example, as discussed earlier, the public concern for animal welfare is focused on well-being as a broad concept encompassing mental health, behavior and positive experiences (Sørensen and Fraser, 2010; Coleman et al., 2016; Buddle et al., 2018), while for producers this appears to relate more to health and disease (Te Velde et al., 2002; Bock and van Huik, 2007; Vanhonacker et al., 2008; Spooner et al., 2012).

There were some significant gender effects on the attitude, knowledge and trust variables as well as some significant interactions. Gender differences regarding concern for animals and their welfare have been reported previously, with females showing greater concern when compared to males (Herzog et al., 1991; Eldridge and Gluck, 1996; Knight et al., 2004; Taylor and Signal, 2005; Randler et al., 2021), including with regard to farm animal welfare (Vanhonacker et al., 2007; Doughty et al., 2017). Some of the notable gender effects in the current study were that males reported more positive beliefs about the conditions provided for sheep and beef cattle during sea and land transport and the husbandry practices used in the red meat industry, while females placed more importance on general welfare attributes. This is consistent with previous research by Doughty et al. (2017) who reported greater concern for sheep welfare in female respondents when compared to male respondents; female members of the public rated sheep welfare significantly worse than male respondents and also placed greater importance on welfare than males. Recent surveys of the Australian public found that females engage in more community behaviors to display dissatisfaction with the way livestock animal are treated than males (Coleman et al., 2015, 2018).

This study helps to quantify the differences between the general public and producer attitudes to welfare issues in the red meat industries, thereby augmenting the findings from previous qualitative research. These results and others from the same group (Coleman and Toukhsati, 2006; Coleman et al., 2016, 2018) indicate a polarization between the public and livestock producers in their attitudes toward animal welfare, knowledge of husbandry practices and trust in each other. The public is a key driver of animal welfare change and thus there needs to be transparency between the livestock producers and the public regarding farming techniques and a clear articulation of the implications for both food quality on the one hand and animal welfare on the other. However, industry responses need to have a balance between listening to community requirements and a preparedness to defend a practice if, on balance, it is considered the best in terms of healthy food, economics and welfare (Coleman, 2010). The results of the present study provide insights into the specific content that would be useful in developing and testing the use of engagement strategies between the public and red meat producers to reduce this polarization, that is, in achieving a degree of convergence between the general public and red meat producers in their attitudes to husbandry practices in the red meat industry, a greater awareness of each group’s concerns and improved trust in livestock people.

There are some limitations to this study that may have impacted on the interpretation of the results. While data from both samples were collected using telephone interviews, the recruitment of participants was random for the general population, but was only random within the available databases of producers. These databases may not have captured producers who, for a variety of reasons, may not have participated in industry activities. The producers who were not accessed may, for example, have been less willing to engage in regular industry forums and may have been less open to seeking new knowledge. Their attitudes may have been more conservative in relation to current practices that those who were recruited for this study. However, given that the producers surveyed here were already more conservative than the general population, including these other producers would have been expected to amplify the results reported here. A second limitation arises from the fact that all data are based on self-reports. To establish the relevance of the attitudes to community behaviors, it would be desirable to conduct experimental research to observe behavioral change following attitude change. While possible, this is a complex and expensive exercise.

## Data availability statement

The datasets presented in this article are not readily available because the human ethics approval for this project does not allow for the datasets to be made publicly available. Requests to access the datasets should be directed to corresponding author.

## Ethics statement

The studies involving human participants were reviewed and approved by the Human Research Ethics Committee at the University of Melbourne. Written informed consent for participation was not required for this study in accordance with the national legislation and the institutional requirements.

## Author contributions

GC, PH, LH, CM, and MR contributed to the conception and design of the study. MR coordinated the participant recruitment and organized and collated the data. GC, LH, and MR performed the statistical analysis. PH and MR wrote the first draft of the manuscript. PH, GC, LH, and MR wrote sections of the manuscript. All authors contributed to the manuscript revision, read, and approved the submitted version.
